# Luteinizing hormone-induced Akt phosphorylation and androgen production are modulated by MAP Kinase in bovine theca cells

**DOI:** 10.1186/1757-2215-2-17

**Published:** 2009-11-16

**Authors:** Shin Fukuda, Makoto Orisaka, Kimihisa Tajima, Katsushige Hattori, Fumikazu Kotsuji

**Affiliations:** 1 Department of Obstetrics & Gynecology, University of Fukui, Matsuoka, Fukui, 910-1193, Japan; 2 University of Fukui, 23-3 Shimoaiduki, Matsuoka, Eiheiji-cho, Yoshida-gun, Fukui, 910-1193, Japan

## Abstract

**Background:**

Theca cells play an important role in controlling ovarian steroidogenesis by providing aromatizable androgens for granulosa cell estrogen biosynthesis. Although it is well established that the steroidogenic activity of theca cells is mainly regulated by LH, the intracellular signal transduction mechanisms that regulate thecal proliferation and/or steroidogenesis remain obscure. In this study, we examined whether and how LH controls the PI3K/Akt signaling pathway and androgen production in bovine theca cells. We also explored whether this LH-induced PI3K/Akt activation is modulated with other signaling pathways (i.e. PKA and MAPK).

**Methods:**

Ovarian theca cells were isolated from bovine small antral follicles and were incubated with LH for various durations. Phospho-Akt and total-Akt content in the cultured theca cells were examined using Western blotting. Androstenedione levels in the spent media were determined using EIA. Semi-quantitative RT-PCR analyses were conducted to analyze the mRNA levels of CYP17A1 and StAR in the theca cells. To examine whether Akt activity is involved in theca cell androgen production, the PI3K inhibitors wortmannin and LY294002 were also added to the cells.

**Results:**

Akt is constitutively expressed, but is gradually phosphorylated in cultured bovine theca cells through exposure to LH. LH significantly increased androstenedione production in bovine theca cells, whereas addition of the wortmannin and LY294002 significantly decreased LH-induced androstenedione production. LH significantly increased CYP17A1 mRNA level in theca cells, whereas addition of LY294002 significantly decreased LH-induced CYP17A1 expression. Neither LH nor PI3K inhibitors alter the mRNA levels of StAR in theca cells. Although H89 (a selective inhibitor of PKA) does not affect LH-mediated changes in Akt, U0126 (a potent MEK inhibitor) suppressed LH-induced Akt phosphorylation, CYP17A1 expression, and androgen production in theca cells.

**Conclusion:**

These results indicate that LH stimulates CYP17 mRNA expression and androgen production in theca cells via activation of the PI3K/Akt pathway. The LH-induced Akt phosphorylation and androgen production are modulated by the MAPK signaling in bovine theca cells.

## Background

The principal function of ovarian theca cells is steroid hormone production. Theca cells play an important role in controlling ovarian steroidogenesis by providing aromatizable androgens for granulosa cell estrogen biosynthesis [1]. Androgens also function as local regulators of ovarian folliculogenesis upon binding androgen receptors localized to granulosa cells, stromal cells, and oocytes [2]. Androgen receptor null mice culminate in reduced fertility and premature ovarian failure [3], indicating that androgens are necessary for reproductive function and fertility. Normal ovarian function requires accurate regulation of steroidogenic activity of theca cells through extraovarian and intraovarian mechanisms. Thecal steroidogenic hyperactivity can cause ovarian dysfunction, such as polycystic ovary syndrome (PCOS) [4].

It is well established that theca cell steroidogenesis is under the primary control of luteinizing hormone (LH) through the second-messenger cAMP-protein kinase A (PKA) pathway [5,6]. Moreover, LH stimulates theca cells to produce androgens and to maintain progesterone production by the induction of genes involved in steroidogenesis: cytochrome P450 side-chain cleavage enzyme (CYP11A1), 3β-hydroxysteroid dehydrogenase, 17α-hydroxylase/C17-20 lyase cytochrome P450 (CYP17A1), and steroidogenic acute regulatory protein (StAR) [7-10].

Intracellular signaling mechanisms that regulate ovarian follicular development and/or steroidogenesis remain obscure [11]. Nevertheless, LH reportedly activates the extracellular-signal-regulated kinases (ERK)/mitogen activated protein kinase (MAPK) pathway in ovarian granulosa and theca cells [12]. Although FSH and several growth factors are known to activate the phosphatidylinositol 3' kinase (PI3K)/Akt pathway in granulosa cells [13-15], whether LH stimulates the PI3K/Akt cascade in theca cells is not clear. Although LH augments androgen production in theca cells, it remains unknown whether this response is mediated via activation of the PI3K/Akt pathway.

In this study, we examined whether and by what means LH controls PI3K/Akt signaling and androgen production using cultured bovine theca cells. We demonstrated that LH stimulates CYP17A1 mRNA expression and androgen production in theca cells via activation of the PI3K pathway. Both the PI3K and the MAPK pathways coordinately regulate androgen production in bovine theca cells.

## Methods

### Exprimental design

#### Experiment 1

To examine whether LH stimulates PI3K/Akt signaling in theca cells, bovine theca cells from small antral follicles were incubated with LH for various durations (0, 5 min, 20 min, 1 h, 2 h, 4 h, 6 h, 8 h, 12 h, 24 h, and 48 h), and phospho-Akt and total-Akt content were examined using Western blotting.

#### Experiment 2

To examine whether Akt activity is involved in theca cell androgen production, theca cells were pretreated for 30 min with the PI3K inhibitors, wortmannin (0.1 μM) and LY294002 (25 μM). The cells were subsequently stimulated with LH (100 ng/ml) for 24 h. Androstenedione levels in the spent media were determined using EIA.

#### Experiment 3

Along with examining androstenedione production, semi-quantitative RT-PCR analyses were conducted to analyze the mRNA levels of CYP17A1 and StAR in the cultured theca cells at 12 h of incubation.

#### Experiment 4

Whether PKA or MAPK pathway influence LH-induced Akt phosphorylation in theca cells was explored. Theca cells were pretreated with H89 (i.e. a selective inhibitor of PKA [16]), and U0126 (i.e. a potent MEK inhibitor) for 30 min. The cells were subsequently stimulated with LH (100 ng/ml) for 24 h. Phospho-Akt and total-Akt content in the cultured theca cells were examined using Western blot at 24 h of the culture. CYP17A1 mRNA levels in the theca cells and androstenedione levels in the spent media were also determined.

### Antibodies

Rabbit polyclonal anti-phospho-Akt (*i.e*. active Akt) antibodies and anti-total-Akt antibodies were purchased from Cell Signaling Technologies (Beverly, MA). Goat anti-rabbit IgG coupled to horseradish peroxidase was purchased from Santa Cruz Biotechnology, Inc. (Santa Cruz, CA).

### Reagents

Human LH was provided by the National Institutes of Health and Dr. A. F. Parlow (National Hormone and Peptide Program, Torrance, CA). LY294002 (a PI3K inhibitor) was from Sigma Chemical Co. (St. Louis, MO), and wortmannin (a PI3K inhibitor), H89 (a selective inhibitor of PKA), and U0126 (a potent MEK inhibitor) were purchased from Calbiochem Novabiochem Corp. (San Diego, CA).

### Theca cell culture

Bovine ovaries were collected less than 15 min after slaughter at a local abattoir. The ovaries were placed in an ice-cold buffered salt solution and transferred to the laboratory less than 90 min after collection. The estrous cycle stage was determined morphologically, as described previously by Ireland *et al *[17]; only those ovaries with a regressing corpus luteum were used for this study. Theca cells were isolated from the ovaries under sterile conditions, as described previously [18]. Briefly, small antral follicles (2-4 mm diameter) with clear surfaces were cut into halves and theca interna removed *in situ *using fine forceps. Granulosa cells, together with part of the theca cell layer, were removed by scraping with a scalpel under a stereomicroscope. The resultant thin thecal layer was minced and subsequently treated with a Hanks'-HEPES buffer containing collagenase (2150 U/ml, type 1; Sigma) and DNase (100 U/ml; Sigma), 0.4% (vol/vol) BSA, and 0.2% (wt/vol) glucose (pH 7.4). Cell dissociation was allowed to continue for 30-60 min at 37°C with continuous stirring at 80 rpm and 0.25% (wt/vol) pancreatin (Sigma) in a Hanks'-HEPES buffer for 7 min. Dispersed cells were washed three times. Cell viability, as determined using the trypan blue-dye exclusion test, was 90-93%. Purity of the theca cell preparation used in this study was substantiated by the secretion of estradiol; prepared theca cells did not produce estradiol in the presence or absence of forskolin, whereas granulosa cells obtained from the same follicle secret significant (data not shown). Isolated theca cells were plated onto serum-coated dishes with serum-free medium for 36 h. Then they were stimulated with LH (100 ng/ml) for various durations (0, 5 min, 20 min, 1 h, 2 h, 4 h, 6 h, 8 h, 12 h, 24 h, and 48 h). Preliminary data indicated that 100 ng/ml of LH is the minimal effective concentration for inducing a significant increase in androgen production and CYP17A1 expression in our culture system.

### Western blot analysis

Western blot analysis was conducted as described previously [12]. Briefly, primary cultures at the end of incubation with the appropriate stimulant or no stimulation as indicated in each experiment were rinsed with ice-cold PBS and once with buffer A [50 mM β-glycerophosphate (pH 7.3), 1.5 mM EGTA, 1 mM EDTA, 1 mM dithiothreitol, and 0.1 mM sodium vanadate] and were subsequently harvested in buffer A plus proteinase inhibitors. Cell lysates were centrifuged at 20,000 × *g *for 20 min. The supernatant was assayed for protein content and subjected to Western blot analysis to detect anti-phospho-Akt and anti-total-Akt. Samples containing equal amounts of protein (40 μg) were separated by 10% acrylamide SDS-PAGE. The relevant proteins were detected on blots using their specific antibodies.

### Determination of androstenedione levels

Androstenedione levels were determined using EIA at the end of the stimulation. Protein was quantified using the Bradford method.

### RNA extraction and RT-PCR

Total RNA was isolated using TRIzol (Invitrogen Corp., Carlsbad, CA) according to the manufacturer's instructions. The RNA pellets were ethanol precipitated, washed, and resuspended in sterile ribonuclease-free water. Quality of the RNA was assessed by fractionating it on 1% agarose gel and observing the presence of the typical 28S and 18S rRNA under UV light. RT-PCR analyses for bovine CYP17A1, StAR, and 36B4 (an acidic ribosomal phosphoprotein as an internal control) were performed on total RNAs from cultured theca cells using specific primers. Primers used for bovine CYP17A1 were 5'-TCAGAGAAGTGCTCCGAATCC-3' and 5'-TGCCACTCCTTCTCACTGTGA-3'; those for bovine StAR were 5'-TCGCGGCTCTCTCCTAGGT-3' and 5'-CTGCCGGCTCTCCTTCTTC-3', and those for bovine 36B4 were 5'-GGCGACCTGGAAGTCCAACT-3' and 5'-GGATCTGCTGCATCTGCTTG-3', respectively. In each case, RNAs were reverse transcribed in a final volume of 40 μl solution containing 1× first-strand buffer [3 mM MgCl_2_, 75 mM KCl, 50 mM Tris-HCl (pH 8.3)], 500 μM each deoxynucleotide triphosphate, 10 mM dithiothreitol, 200 U SuperScript III RNase H-free reverse transcriptase (Invitrogen Corp.), 200 ng random hexamers, and 2 μg total RNA. The target cDNAs were amplified for 30 cycles (CYP17A1 and StAR) and 25 cycles (36B4, internal control), respectively, in a thermal cycler (94 C for 20 s, 60 C for 30 s, and 72 C for 60 s) using deoxynucleotide triphosphate (0.2 mM) and 1.5 U of TaKaRa Ex Taq (Takara Shuzo Co. Ltd., Kyoto, Japan). Aliquots of PCR products were electrophoresed on 1.5% agarose gels and stained with ethidium bromide. The relative integrated density of each band was scanned and digitized using FluorChem (Alpha Innotech Corporation, San Leandro, CA); the ratios of densitometric readings of the amplified target cDNA and internal control, 36B4, DNA were analyzed.

### Statistical analysis

All experiments were repeated at least three times using theca cells obtained from separate groups of bovines. Data were subjected to ANOVA. Group means were contrasted using Tukey's *post hoc *multiple comparison test. *P *< 0.05 was considered significant. All values are expressed as mean ± SEM.

## Results

### Experiment 1

#### LH increases phospho-Akt content in bovine theca cells

**Figure 1:**
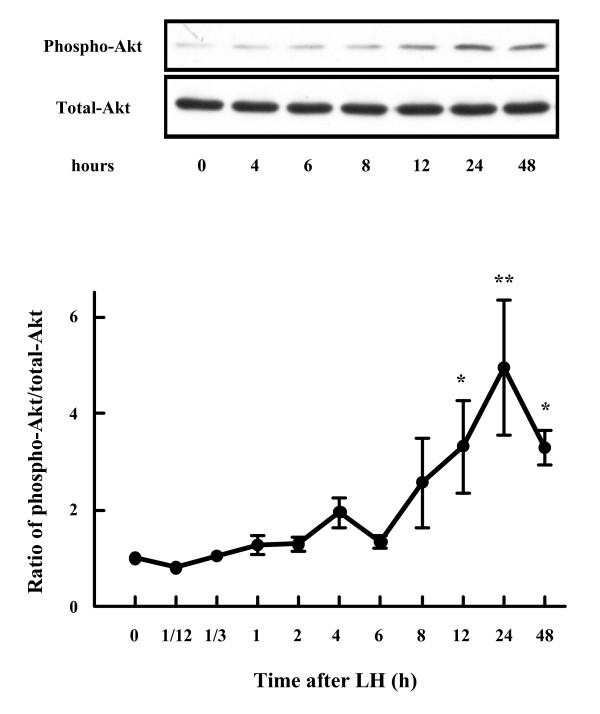
**Time-course effect of LH on Akt phosphorylation in bovine theca cells**. Theca cells were plated onto serum-coated dishes with serum-free medium for 36 h and then stimulated with LH (100 ng/ml) for the stated times. Cytosolic extracts (20 μg) were subjected to immunoblotting with anti-phosphorylated-Akt antibody and anti-total-Akt antibody. Representative images (*Top*) and densitometric data of phospho-Akt contents (*Bottom*), expressed as ratio of phospho-Akt to total-Akt, are shown. * denotes means that are significantly different from 0 h (*P *< 0.01). ** denotes means that are significantly different from 0 h (*P *< 0.001).

Total-Akt was present in theca cells at 0 h and remained constant during culture with LH. During the 5 min to 8 h of culture, Akt was not phosphorylated by LH. However, the amount of phospho-Akt began to increase at 12 h and reached its highest level (five-fold higher than baseline) at 24 h after addition of LH (Fig. 1).

### Experiment 2

#### Effects of the PI3K inhibitors on LH-induced androgen production in theca cells

**Figure 2:**
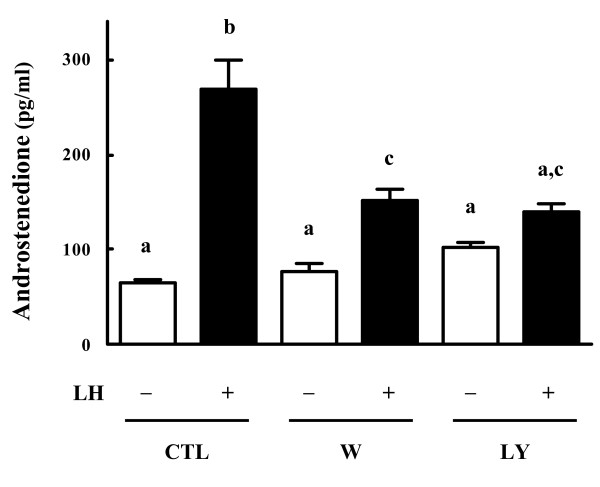
**Effects of PI3K inhibitors on androstenedione production in bovine theca cells**. Bovine theca cells were stimulated with LH (100 ng/ml), wortmannin (W, 0.1 μM), LY294002 (LY, 25 μM), or their combination for 24 h in serum-coated dishes with serum-free medium. Control cells (CTL) were cultured in the absence of added treatments. Culture media were assayed for androstenedione by EIA. Values are means ± SEM for four experiments. *Different letters *denote a significant difference of means (*P *< 0.05).

Results show that LH significantly increased androstenedione production in bovine theca cells. Addition of the PI3K inhibitors wortmannin and LY294002 significantly decreased LH-induced androstenedione production in theca cells (Fig. 2).

### Experiment 3

#### Effects of the PI3K inhibitors on CYP17 and StAR mRNA expressions in theca cells

**Figure 3:**
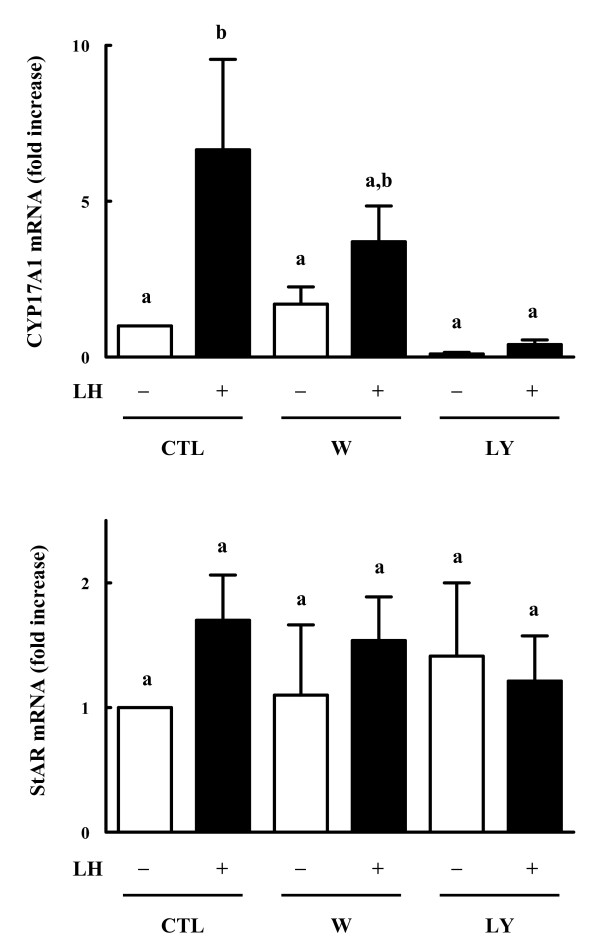
**Effects of PI3K inhibitors on mRNA expression of CYP17A1 and StAR in bovine theca cells**. Theca cells were incubated with LH in the presence or absence of wortmannin (0.1 μM) or LY294002 (25 μM) in serum-coated dishes with serum free medium for 12 h. Control cells (CTL) were cultured in the absence of added treatments. Then RT-PCR was conducted using CYP17A1, StAR, and 36B4 (internal control) primers using total RNA isolated from the cells. The products were fractionated on 1% agarose gel and stained with ethidium bromide. The mRNA levels of CYP17A1 and StAR were expressed as ratio to 36B4 values. Data are the mean ± SEM (*n *= 5). *Different letters *represent statistically significant differences of means (*P *< 0.05).

Results show that LH significantly increased CYP17A1 mRNA level in the theca cells. Addition of LY294002, but not wortmannin, significantly decreased LH-induced CYP17A1 mRNA expression (Fig. 3). Neither LH nor the PI3K inhibitors alter the mRNA levels of StAR in the theca cells.

### Experiment 4

#### Effect of PKA inhibitor and MEK inhibitor on LH-induced Akt phosphorylation

**Figure 4:**
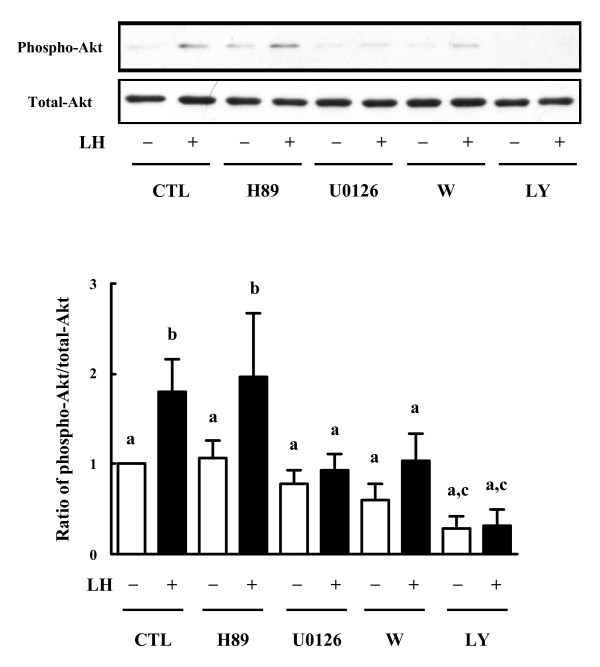
**Effects of PKA inhibitor, MEK inhibitor and PI3K inhibitors on Akt phosphorylation in bovine theca cells**. Subconfluent cultures were pretreated with PKA inhibitor (H89, 3 μM), MEK inhibitor (U0126, 10 μM), or PI3K inhibitors (wortmannin, 0.1 μM; LY294002, 25 μM) for 30 min. Then they were stimulated with LH (100 ng/ml) for 24 h. Control cells (CTL) were cultured in the absence of added treatments. Cell lysates (20 μg) were subjected to SDS-PAGE and Western blot using anti-phosphorylated-Akt antibody (Phospho-Akt) or anti-total-Akt antibody (Total-Akt). Representative images (*Top*) and densitometric data of phospho-Akt contents (*Bottom*), expressed as a ratio of phospho-Akt to total-Akt, are shown. Values show the mean ± SEM for three experiments. Each experiment was reproduced at least three times. *Different letters *denote significant differences of means (*P *< 0.05).

In fact, H89 (i.e. a selective inhibitor of PKA) did not affect LH-mediated changes in Akt. On the other hand, U0126 (i.e. a potent MEK inhibitor) inhibited LH-induced Akt phosphorylation in the theca cells (Fig. 4).

**Figure 5:**
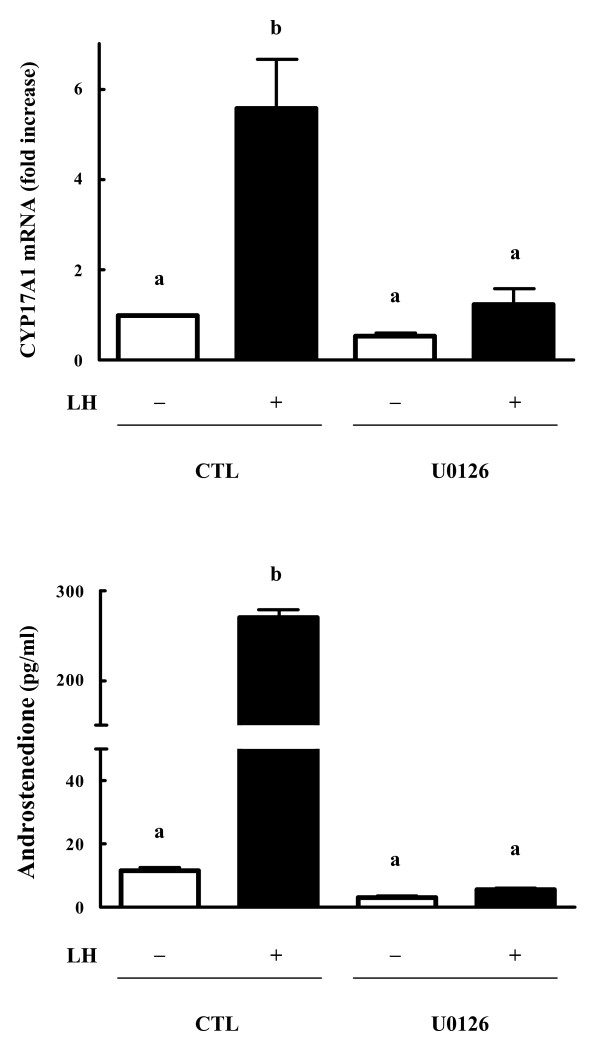
**Effects of MEK inhibitor on CYP17A1 mRNA expression and androstenedione production in bovine theca cells**. Subconfluent cultures were pretreated with MEK inhibitor (U0126, 10 μM) for 30 min. Then they were stimulated with LH (100 ng/ml) for 12-24 h. Control cells (CTL) were cultured in the absence of added treatments. RT-PCR was conducted using CYP17A1 and 36B4 (internal control) primers using total RNA isolated from the cells. The mRNA level of CYP17A1 were expressed as ratio to 36B4 values (*Top*). Culture media were also assayed for androstenedione by EIA (*Bottom*). Data are the mean ± SEM (*n *= 4). Each experiment was reproduced at least three times. *Different letters *represent statistically significant differences of means (*P *< 0.05).

Although LH stimulated CYP17A1 mRNA expression and androstenedione production in the theca cells, the MAPK cascade inhibitor (U0126) completely blocked these responses (Fig. 5).

## Discussion

In this study, we demonstrated that: 1) Akt is constitutively expressed, but is gradually phosphorylated in cultured bovine theca cells through exposure to LH; 2) LH stimulated androstenedione production in theca cells, although addition of the PI3K inhibitors (i.e. wortmannin and LY294002) attenuated LH-induced androstenedione production; 3) LH increased CYP17A1 mRNA level in theca cells, whereas addition of LY294002 suppressed LH-induced CYP17A1 expression in theca cells; 4) although H89 (i.e. a selective inhibitor of PKA) did not affect LH-mediated changes in Akt, U0126 (i.e. a potent MEK inhibitor) inhibited the LH-induced Akt phosphorylation, CYP17A1 expression, and androgen production in theca cells. These results suggest that LH stimulates CYP17A1 mRNA expression and androgen production in theca cells via activation of the PI3K/Akt pathway, and that the MAPK, not PKA, is involved in LH stimulation of the PI3K/Akt cascade in bovine theca cells.

PI3K converts phosphatidylinositol-4,5-biphosphate to phosphatidylinositol-3,4,5-triphosphate, leading to activation of downstream kinases including Akt, which in turn phosphorylates Bad, forkhead in rhabdomyosarcoma (FKHR), Fas-associated death domain-like IL-1β-converting enzyme-like inhibitory protein (FLIP), and X-linked inhibitor of apoptosis protein (XIAP) [19]. The PI3K/Akt activation drives cell through many biological functions, including gene expression, cell cycle, survival, glucidic metabolism, endocytosis and vesicular trafficking, cell transformation, and oncogenesis [20]. In ovary, FSH and several growth factors are known to activate the PI3K/Akt pathway and prevent apoptosis in granulosa cells and cultured follicles [13-15]. Although LH has been reported to activate the cAMP/PKA pathway [4] and the ERK/MAPK pathway [12] in theca cells, whether LH stimulates the PI3K/Akt cascade in theca cells remains unclear. Results of this study show for the first time that 1) LH stimulates Akt phosphorylation in cultured bovine theca cells, and that 2) activation of PI3K/Akt is involved in CYP17A1 mRNA expression and androgen production in theca cells. Reportedly, LH induced Akt phosphorylation in whole rat ovary [21], and the PI3K inhibitor, LY294002, suppressed androstenedione production by theca cells in rat [22] and cattle [11]. It is possible that LH-stimulated Akt phosphorylation in theca cells is responsible for these observations reported earlier.

Both wortmannin and LY294002 are inhibitors of the lipid-modifying enzymes known as PI3K, and many researchers perform a parallel study by using both inhibitors to probe the roles of PI3K in biological processes. However, depending on the concentration examined, these inhibitors could be non-specific and cytotoxic and could complicate the interpretation of their findings. In our system, the 0.1 μM of wortmannin and 25 μM of LY294002 are the minimal effective concentrations for blocking the LH-induced androstenedione production in theca cells. Nevertheless, only LY294002 suppressed LH-induced CYP17A1 mRNA expression, whereas wortmannin did not affect this response. While the reason for this apparent discrepancy is not clear, it is worth noting that wortmannin has been reported to be unstable in aqueous solution and less specific than LY294002 [23,24]. Higher concentration (> 0.1 μM) of wortmannin induced theca cell detachment and apoptosis in our serum-free culture system.

Numerous reports have described that an activation of the intracellular signaling (i.e. cAMP/PKA, ERK/MAPK, and PI3K/Akt) is a rapid reaction in most cells. However, in this study, it took 12 h for LH-induced increase in phospho-Akt content in theca cells. It is of interest whether PKA pathway, which is considered to be a major mediator of the LH-generated signaling, and/or the MAPK pathway influence LH-induced Akt phosphorylation or not. *Experiment 4 *was performed to verify this point.

As described earlier, H89, a potent and selective inhibitor of PKA, did not affect LH-mediated changes in phospho-Akt, indicating that a pathway distinct from that of PKA is involved in LH-induced Akt phosphorylation in theca cells. Until recently, the effects of cAMP were generally thought to be mediated by activation of cAMP-dependent PKA, a major cAMP target, followed by phosphorylation of many intracellular targets, such as cAMP responsive element binding protein (CREB) [25], resulting in changes in ovarian gene expression such as CYP17A1. Nevertheless, some effects of cAMP appear to be inexplicable by activation of PKA. For instance, TSH and cAMP regulate proliferation of thyroid cells by mechanisms independent of PKA [26-29]. Actually, cAMP binds specific guanine nucleotide exchange factors: cAMP-GEFs (also called exchange protein activated by cAMP, Epac) [30,31]. Gonzalez-Robayna *et al*. reported that cAMP-GEFs are expressed in rat granulosa cells and that the cAMP-GEFs play a role in FSH-induced activation of the PI3K/Akt pathway in granulosa cells by PKA-independent manner [32]. Whether theca cells also express these regulatory components and whether the (PKA-independent) cAMP-GEFs mechanism is involved in LH-induced Akt phosphorylation in theca cells remains to be elucidated.

In contrast to PKA inhibitor, the MEK inhibitor (U0126) blocked LH-mediated Akt phosphorylation and androgen production in theca cells. Reportedly, the MAPK inhibitor also inhibits FSH-mediated Akt phosphorylation in rat granulosa cells [32]. While the precise mechanism for the activation of PI3K pathway by LH in theca cells is not known, it is possible that the LH-induced phospho-Akt up-regulation may involve MAPK-mediated down-regulation of phosphatase and tensin homologue (PTEN; a tumor suppressor which negatively regulates Akt phosphorylation). In this context, it has been shown that PI3K is required for estradiol-stimulated hepatic cell growth and that the MAPK pathway reduces the level of PTEN, allowing estradiol-induced phosphorylation of Akt [20]. Whether this indeed is the case in the theca cells awaits further investigation.

As a mechanism explaining why phospho-Akt content in theca cells was increased only after 12 h of incubation with LH, we are also interested in autocrine effects of insulin-like growth factor-II (IGF-II) and nerve growth factor (NGF) on theca cells. Reportedly, theca cells express IGF-II and NGF in cattle, and each of IGF-II and NGF stimulate androgen production [33,34]. Whether LH induces gene/protein expression of these growth factors, and whether it modulates the LH-mediated Akt phosphorylation in theca cells, are subjects that are currently under investigation in our laboratory.

## Conclusion

Taking this evidence together, we conclude that LH stimulates CYP17A1 mRNA expression and androgen production in theca cells via activation of the PI3K/Akt pathway. LH acts in theca cells by PKA-independent mechanisms as well as PKA-dependent mechanisms, each of which controls androgen production. Both the PI3K and the MAPK pathways coordinately regulate androgen production in bovine theca cells. Clarification of the LH-mediated intracellular signaling events is essential for better understanding of not only ovarian physiology, but also of the pathophysiology of PCOS.

## Abbreviations

LH: luteinizing hormone; cAMP: cyclic adenosine monophosphate; PKA: protein kinase A; CYP17A1: 17α-hydroxylase/C17-20 lyase cytochrome P450; StAR: steroidogenic acute regulatory protein; ERK: extracellular-signal regulated kinase; MAPK: mitogen activated protein kinase; PI3K: phosphatidyl inositol 3-kinase; EIA: enzyme immunoassay; RT-PCR: reverse transcription polymerase chain reaction; MEK: MAPK/ERK kinase; 36B4: acidic ribosomal phosphoprotein; GEFs: guanine nucleotide exchange factors; PTEN: phosphatase and tensin homologue; PCOS: polycystic ovary syndrome.

## Competing interests

The authors declare that they have no competing interests.

## Authors' contributions

SF, MO, KT, KH, and FK conceived of the study, participated in its design and coordination and drafted the manuscript. All authors read and approved the final version of the manuscript.
